# Infection with *Leishmania major* Induces a Cellular Stress Response in Macrophages

**DOI:** 10.1371/journal.pone.0085715

**Published:** 2014-01-09

**Authors:** Alessandra A. Filardy, Ana Caroline Costa-da-Silva, Carolina M. Koeller, Kamila Guimarães-Pinto, Flávia L. Ribeiro-Gomes, Marcela F. Lopes, Norton Heise, Célio G. Freire-de-Lima, Marise P. Nunes, George A. DosReis

**Affiliations:** 1 Carlos Chagas Filho Institute of Biophysics, Federal University of Rio de Janeiro, Rio de Janeiro, Brazil; 2 Laboratory of Parasitic Diseases, National Institutes of Health, Bethesda, Maryland, United States of America; 3 Oswaldo Cruz Institute, Oswaldo Cruz Foundation, Rio de Janeiro, Brazil; Federal University of São Paulo, Brazil

## Abstract

We investigated early cellular responses induced by infection with *Leishmania major* in macrophages from resistant C57/BL6 mice. Infection increased production of reactive oxygen species by resident, but not inflammatory peritoneal macrophages. In addition, infection increased activation of stress-activated protein kinases/c-Jun N-terminal kinases (SAPK/JNK) in resident, but not in inflammatory peritoneal macrophages. Infection also increased expression of membrane and soluble FasL, but infected macrophages remained viable after 48 h. Infection increased secretion of cytokines/chemokines TNF-α, IL-6, TIMP-1, IL-1RA, G-CSF, TREM, KC, MIP-1α, MIP-1β, MCP-1, and MIP-2 in resident macrophages. Addition of antioxidants deferoxamine and N-acetylcysteine reduced ROS generation and JNK activation. Addition of antioxidants or JNK inhibitor SP600125 reduced secretion of KC. Furthermore, treatment with antioxidants or JNK inhibitor also reduced intracellular parasite replication. These results indicated that infection triggers a rapid cellular stress response in resident macrophages which induces proinflammatory signals, but is also involved in parasite survival and replication in host macrophages.

## Introduction

Infection with *Leishmania* affects nearly 350 million people worldwide. Parasites infect host macrophages and survive as intracellular amastigotes within phagolysosomal vesicles. Both tissue resident and inflammatory macrophages can be infected [1], [2]. Macrophages produce reactive oxygen species (ROS) upon infection with *Leishmania* [3]. Although ROS are regarded as toxic for the parasite, there is increasing evidence that ROS also function as signaling intermediates required for parasite differentiation to amastigotes [4], [5]. In addition, infection with *L. major* induces cytokine and chemokine gene expression in macrophages [6], [7] and recruits an early inflammatory reaction [6]. Subsequent interactions with inflammatory neutrophils either increases or decreases *L. major* replication in macrophages depending on host genotype, and through mechanisms involving either TGF-β or Neutrophil Elastase [8]–[10].

Mammalian cells respond to environmental stress by either adapting or undergoing programmed cell death [11]. Cellular stress activates the intracellular stress-activated protein kinases/c-Jun N-terminal kinases (SAPK/JNK) [11], [12]. Signalling through JNK activates c-Jun/AP-1 and increases expression of the death ligand FasL [13]–[15]. Therefore, cellular responses to stress could result in Fas-mediated apoptosis. However, the JNK pathway is also involved in non-apoptotic responses such as macrophage differentiation [16] and proinflammatory cytokine and chemokine production [17], [18].

Here we investigated early cellular and immunological responses to *L. major* infection in macrophages from genetically resistant mice. Our results indicated that infection triggers a cellular stress response in resident macrophages, characterized by increased production of reactive oxygen species (ROS), activation of the JNK stress pathway, and chemokine production. Addition of antioxidants or JNK inhibitor blocked both chemokine production and parasite replication. These results indicated that activation of macrophages to mediate an inflammatory response is triggered by a stress stimulus provided by the parasite, and mediated by ROS and the JNK signaling pathway.

## Results

### Production of ROS Induced by *L. major* Infection

**Figure 1:**
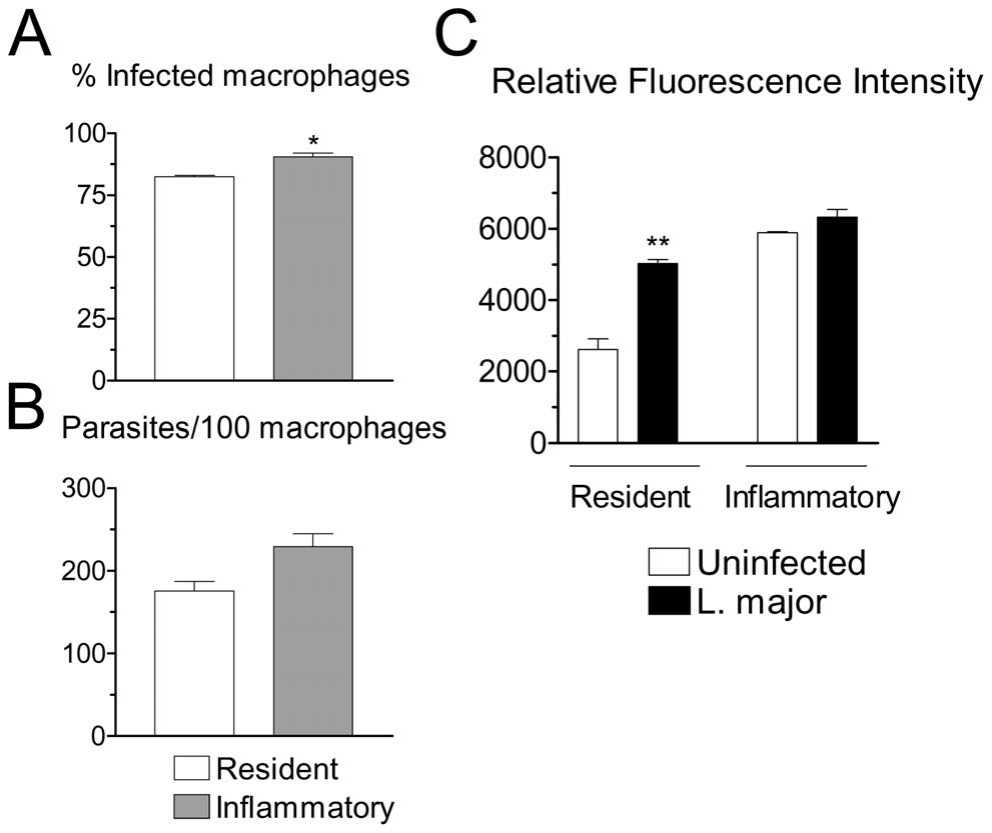
Infection of macrophages with *L. major* and generation of ROS. (A, B) Resident or inflammatory macrophages from B6 mice were infected with *L. major* for 4 h, and washed. Cells were stained and percentages of infected macrophages (A) and number of parasites per 100 macrophages (B) were determined. (C) Resident or inflammatory B6 macrophages were loaded with DCFH-DA, washed, treated with medium (Uninfected) or with *L. major* for 4 h, and fluorescence was measured. Results indicate arbitrary units of fluorescence and are mean and SE of triplicates. *P<0.05; **P<0.01.

Peritoneal resident and inflammatory macrophages from C57BL/6 (B6) mice showed a comparable degree of infection 4 h after interaction with *L. major* promastigotes, in spite of a small, but statistically significant increase in percentage of infected inflammatory cells (Figures 1A and 1B). Infection with *Leishmania* parasites triggers production of ROS by macrophages [3], [19], [20]. We therefore investigated production of ROS 4 h after infection of macrophages with *L. major* promastigotes. In preliminary experiments, this time of infection gave the strongest signal of ROS production for the parasite isolate we employed in the present study. The timing of the peak ROS response depends on the parasite isolate employed. Infection increased the level of ROS produced by resident macrophages (Figure 1C). The levels of ROS produced by inflammatory macrophages were already elevated, and infection resulted in little or no additional increase in ROS production (Figure 1C). These results suggested that resident macrophages undergo a more pronouned oxidative response following infection with *L. major*, compared to inflammatory macrophages. In inflammatory macrophages, however, the levels of ROS were already elevated prior to infection.

### Activation of SAPK/JNK Pathway by *L. major* Infection

**Figure 2:**
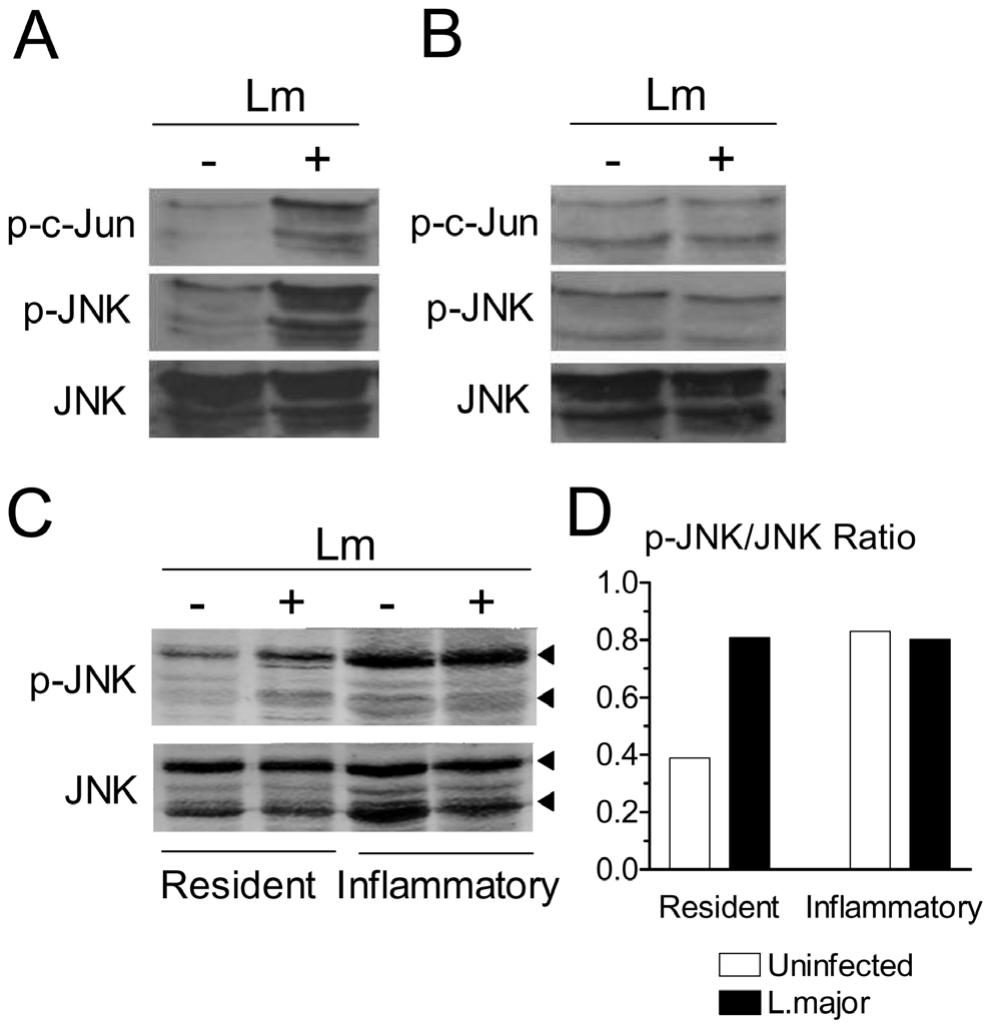
Infection with *L. major* activates the SAPK/JNK pathway. Resident (A) or inflammatory (B) B6 adherent macrophages were infected or not with *L. major* (Lm). After 4 h, cell extracts were obtained and the levels of JNK, p-JNK and p-c-Jun were determined by western blotting. (C) Resident and inflammatory macrophages were adhered and infected in parallel. After 4 h, the levels of JNK and p-JNK were determined by western blotting. Top and bottom arrowheads indicate the p54 and p46 JNK bands, respectively. (D) Densitometric analysis of the blot shown in Figure 2C. The areas of both p46 and p54 bands were scanned. Results were normalized as the ratio between the intensities of the p-JNK and total JNK bands.

Oxidative stress is associated with activation of the SAPK/JNK pathway [21]–[23], where members of the c-Jun family are phosphorylated by JNK [13]–[15]. We investigated the activation of this pathway in macrophages. Western blotting analysis indicated that infection of resident macrophages with *L. major* markedly increased the levels of the phosphorylated forms of c-Jun and JNK over uninfected values (Figure 2A). By densitometric analysis, the increase was 4.1-fold for p-c-Jun, and 2.4-fold for p-JNK. On the other hand, infection induced only a small increase in the levels of p-c-Jun (1.3-fold) and failed to increase p-JNK (0.77-fold) in inflammatory macrophages (Figure 2B). The levels of total JNK protein did not change following infection (Figures 2A and 2B). Anti-p-c-Jun, p-JNK and JNK antibodies reacted with extracts of *Leishmania* promastigotes, but the bands had distinct molecular weight, compared to the mammalian proteins (data not shown). The results shown in Figures 2A and 2B were from independent experiments. We then compared the levels of p-JNK in resident and inflammatory macrophages infected in parallel. Again, infection increased the levels of p-JNK in resident macrophages (Figure 2C). The levels of p-JNK were already elevated in inflammatory macrophages, and did not change following infection (Figure 2C). A densitometric analysis of the blot shown in Figure 2C is presented in Figure 2D and confirms these observations. Infection with purified metacyclic forms also increased the levels of ROS and p-JNK in resident macrophages (not shown). The results indicated that, following infection with *L. major*, resident macrophages initiate a cellular stress response characterized by production of ROS and activation of the SAPK/JNK pathway. On the other hand, inflammatory macrophages were already activated, and infection did not change this ongoing activation state.

### Upregulation of FasL Expression Following *L. major* Infection

**Figure 3:**
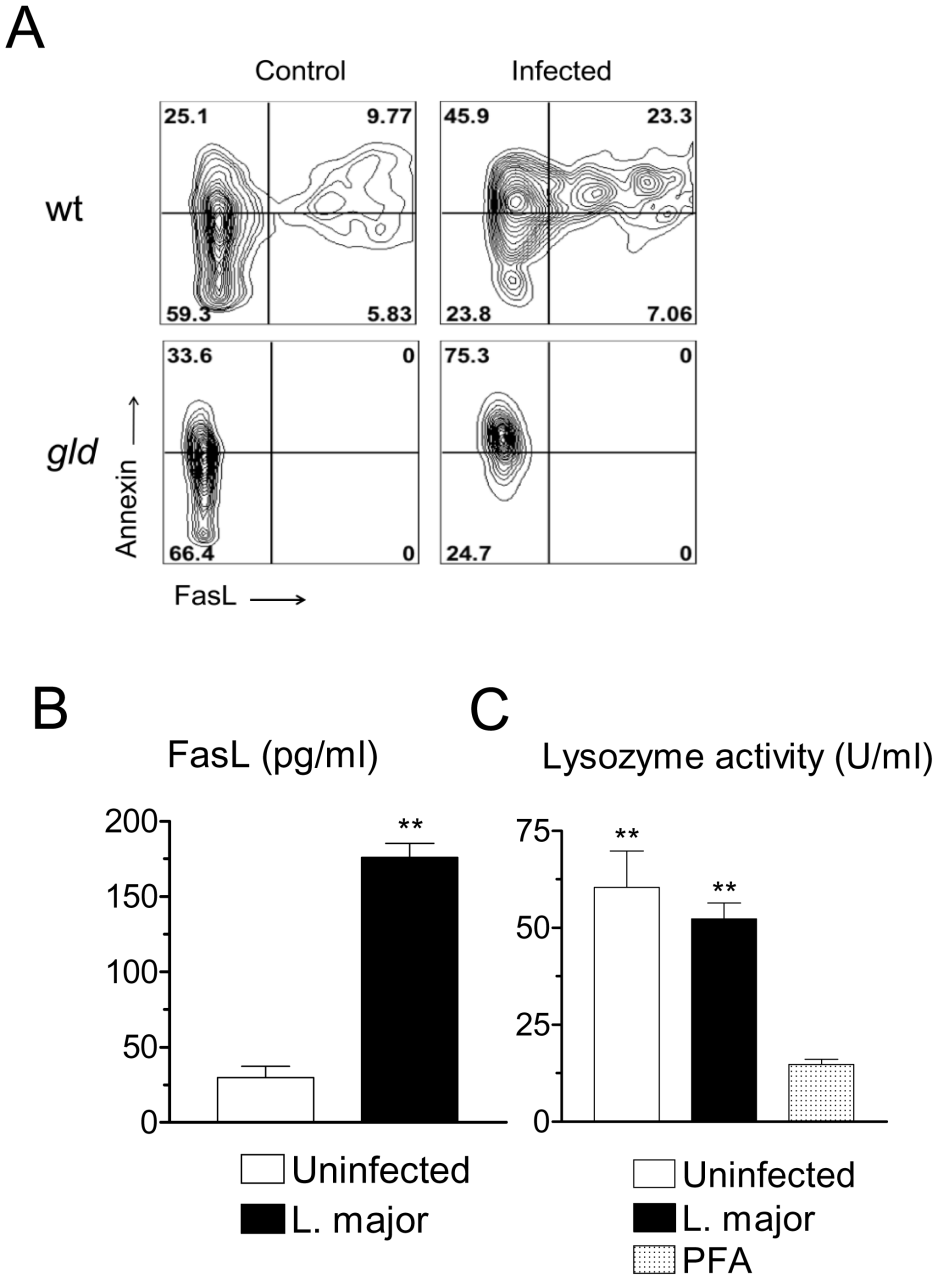
Infection with *L. major* increases FasL expression, but does not induce cell death. (A) Resident B6 macrophages, either wild-type (wt) or FasL-deficient *gld*, were infected or not for 20 h. Monolayers were detached and stained for FCM. Gated populations comprise F4/80^+^ CD11b^+^7AAD^−^ viable macrophages. Results indicate contour plots of Annexin V versus FasL staining. Numbers indicate percentages of cells in each quadrant. (B) Levels of soluble FasL in the supernatants of either control or infected resident macrophages 28 h after infection. (C) Resident macrophages were infected with *L. major* and cultured for 48 h. Supernatants were assayed for lyzozyme activity. As a control, macrophages were treated with paraformaldehyde (PFA) before collecting the supernatant. Results are mean and SE of triplicate cultures. **P<0.01.

Cellular stress responses mediated by the SAPK/JNK pathway can be mediated through Fas and FasL molecules [13]–[15], [24]. We therefore, investigated expression of FasL. Infection increased expression of membrane FasL in resident macrophages (Figure 3A). In agreement with previous reports [25], [26], we found that a proportion of viable macrophages binds Annexin V (Figure 3A). Infection with *L. major* further increased Annexin V binding, including in FasL-deficient *gld* macrophages (Figure 3A). Infection with *L. major* also increased the amount of soluble FasL released by macrophages (Figure 3B). Infected macrophages expressed high levels of Fas receptor (not shown). However, we observed that infected macrophages remained viable and healthy, even after 48 h of culture. A quantitative viability assay based on the constitutive release of lysozyme by macrophages confirmed that, after 48 h, the viability of infected macrophages was comparable to that of uninfected macrophages (Figure 3C).

### Infection Induces a Defined Profile of Cytokine and Chemokine Production by Resident Macrophages

**Figure 4:**
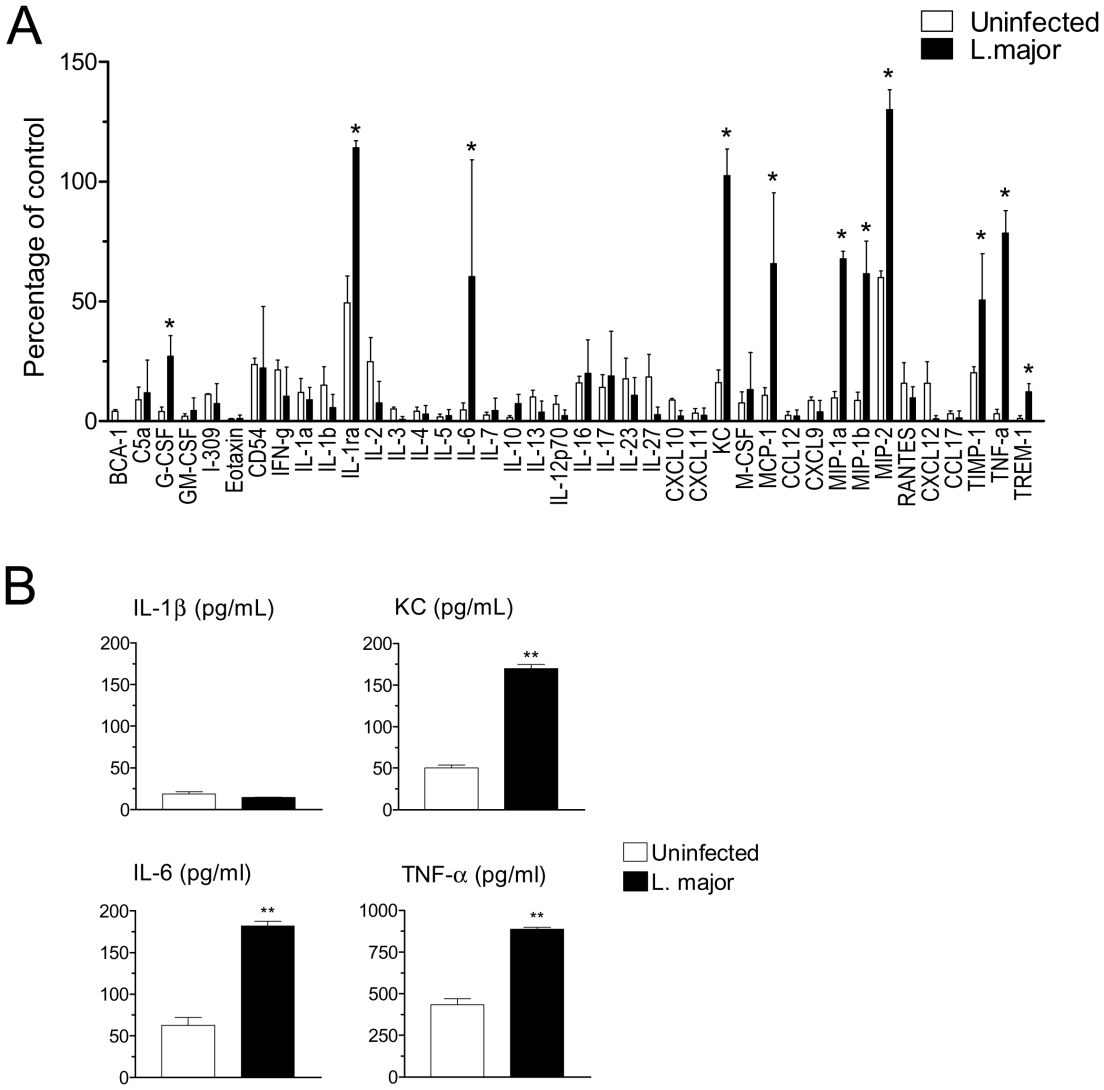
Induction of cytokine and chemokine release by *L. major* infection. (A) Resident B6 macrophages were infected (closed bars) or not (open bars) for 20 h with *L. major*, and supernatants were probed with a mouse cytokine array. The intensity of the labeling for each cytokine/chemokine/mediator was quantitated and normalized as percentage of a positive control provided in the kit. Data indicate mean and SD of two independent arrays. Infected versus uninfected values were compared using non-parametric Mann-Whitney U-test. Cytokines showing a significant (P<0.05) increase following infection are indicated by an asterisk. (B) Supernatants were also probed by ELISA for IL-1β, KC, IL-6 and TNF-α, as indicated. **P<0.01.

We used an antibody array to investigate global cytokine and chemokine production by resident macrophages at the protein level. Following infection with *L. major*, a defined response profile was identified, which could be reproduced in a repeat experiment. Infection increased secretion of cytokines/mediators IL-1RA, IL-6, TNF-α and TIMP-1 (Figure 4A). Infection also increased expression of G-CSF and TREM, although at relatively lower levels. Both IL-1α and IL-1β gave negative results. (Figure 4A). In addition, infection increased the release of chemokines KC, MCP-1, MIP-1α, MIP-1β and MIP-2 (Figure 4A). Individual ELISA assays for IL-1β, KC, IL-6 and TNF-α confirmed the results obtained with the antibody arrays (Figure 4B).

### Regulation of Chemokine Production and Parasite Replication by JNK and ROS

**Figure 5:**
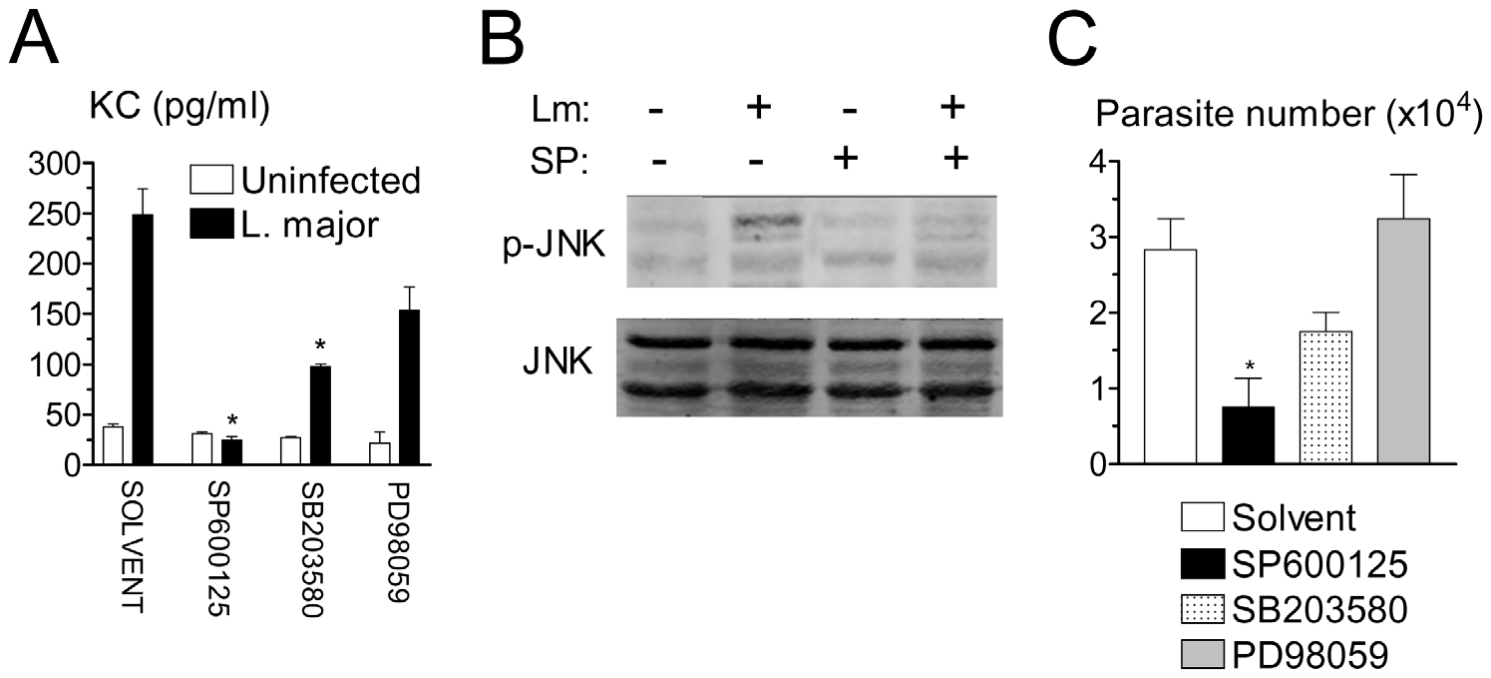
Effect of JNK inhibitor on release of KC, JNK activity and intramacrophagic parasite growth. (A) Resident macrophages were infected or not with *L. major* in the presence of solvent or the indicated MAPK inhibitors. The levels of KC were determined by ELISA after 20 h of infection. (B) Macrophages were infected or not in the presence of solvent or JNK inhibitor SP600125. After 4 h, the levels of JNK and p-JNK were determined by western blotting. (C) Macrophages were infected overnight and cultured for additional 3 d in the presence of solvent or MAPK inhibitors. Intracellular load of parasites was evaluated. Results are mean and SE of the number of extracellular promastigotes produced. *P<0.05, compared to treatment with solvent.

Based on the previous results, we employed secretion of KC as a marker of the inflammatory response of infected macrophages. JNK pathway is involved in inflammatory cytokine and chemokine production [17], [18]. To investigate the role of JNK activity in KC secretion, we employed selective MAPK inhibitors at optimal doses previously determined for peritoneal macrophages [27]. Secretion of KC was completely prevented by JNK inhibitor SP600125 at 10 µM, and partially prevented by ERK inhibitor SB203580 at 5 µM (Figure 5A). Addition of p38 inhibitor PD98059 induced a lesser reduction of KC secretion, and the effect was not statistically significant. We confirmed that treatment of infected macrophages with 10 µM SP600125 blocked JNK activation (Figure 5B). Interestingly, at the same dosages as above, JNK inhibitor SP600125 markedly reduced parasite replication in infected macrophages (Figure 5C), whereas addition of ERK inhibitor SB203580 and p38 inhibitor PD98059 did not result in significant effects (Figure 5C). Previous studies indicate that secretion of KC by infected BALB/c macrophages can be blocked by FasL neutralization [9]. However, secretion of KC induced by infection of B6 macrophages was not inhibited by a blocking mAb specific for FasL or by a neutralizating Fas/Fc chimera (data not shown). In adiition, neutrophil recruitment induced by *L. major* injection was similar in in wild type and FasL-deficient gld B6 mice (not shown). These results suggested that chemokine release and neutrophil recruitment were independent on FasL expression.

**Figure 6:**
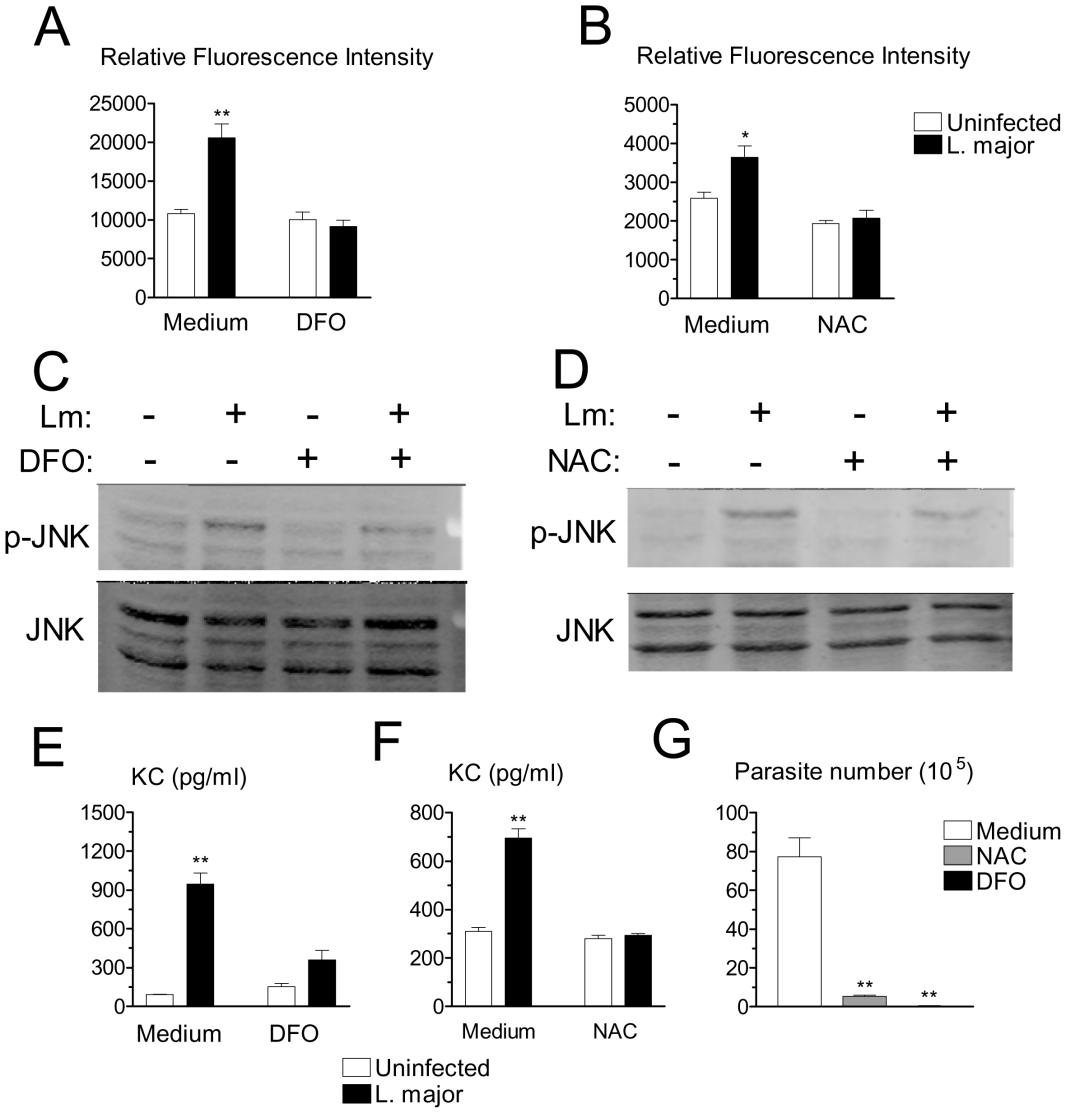
Effects of antioxidants on ROS generation, JNK activation, KC release, and intramacrophagic parasite growth. (A, B) Macrophages were loaded with DCFH-DA, washed and infected or not with *L. major* for 4 h in the presence of medium, antioxidants DFO (A), or NAC (B). Results indicate arbitrary units of fluorescence and are mean and SE of triplicates. (C, D) Macrophages were infected or not in the presence of medium, DFO (C), or NAC (D). After 4 h, the levels of JNK and p-JNK were determined by western blotting. (E, F) Macrophages were infected or not in the presence of medium, DFO (E), or NAC (F). The levels of KC were determined by ELISA after 20 h of infection. (G) Macrophages were infected overnight and cultured for additional 3 d in the presence of medium, DFO or NAC. Intracellular load of parasites was evaluated. Results are mean and SE of extracellular promastigotes produced. *P<0.05, **P<0.01.

JNK activation can be mediated by ROS [21]–[23]. We therefore investigated the effects of antioxidants deferoxamine (DFO), an iron chelator that inhibits radical production, and N-acetylcysteine (NAC), a thiol compound that increases the levels of reduced glutathione. DFO, at 1 mM, and NAC, at 20 mM, markedly attenuated generation of ROS induced by *L. major* infection (Figure 6A and 6B). In addition, DFO and NAC at the same dosages, reduced JNK activation (Figure 6C and 6D) and production of KC induced by *L. major* in macrophages (Figure 6E and 6F). These results suggested that ROS are located upstream of JNK activation. In agreement with the effect of JNK inhibitor, addition of either NAC or DFO at the same dosages as above, markedly suppressed survival/replication of *L. major* in macrophages (Figure 6G). These results indicated important roles for ROS and JNK in both chemokine production and parasite replication in macrophages.

## Discussion

Our results indicated that infection with *L. major* induces a cellular stress response in tissue resident macrophages, characterized by increased ROS generation, SAPK/JNK activation, c-Jun activation, and increased FasL expression. Previous studies indicate that infection with *Leishmania* induces oxidative stress in macrophages [3], [19], [20]. In agreement, our results indicated that infection with *L. major* increased ROS generation in tissue resident macrophages. Inflammatory macrophages, on the other hand, had already increased levels of ROS, which did not increase further upon *Leishmania* infection. Increased production of ROS activates the SAPK/JNK pathway [21]–[23]. In agreement with this notion, our data demonstrated that infection activated JNK and c-Jun in resident macrophages. Infection did not activate, or marginally activated JNK and c-Jun in inflammatory macrophages. However, it should be noted that inflammatory macrophages already expressed elevated levels of ROS and activated JNK prior to infection. Our results disagree with previous studies showing downregulation of MAPK activity induced by *L. major* infection [28]. Perhaps differences in the virulence of the parasite isolate and the use of a retrovirally transformed macrophage cell line in the latter studies [28] could explain the different results.

Cellular stress induces FasL expression through the JNK pathway [13]–[15]. In agreement, our data showed that infection increased expression of both surface and soluble FasL by macrophages. However, our results revealed strain differences in the effects of FasL on infected macrophages. In contrast to FasL-mediated death in BALB/c macrophages [9], infected B6 macrophages remained viable in spite of Fas and FasL expression. Expression of FasL is immunoprotective for *L. major* infection in resistant [29]–[31], but is deleterious in susceptible mice [9]. Furthermore, KC secretion and neutrophil extravasation did not require FasL. Different factors could be involved in these differences. First, BALB/c and B6 FasL molecules express a genetic polymorphism, where BALB/c FasL has greater cytotoxic activity than B6 FasL [32]. Second, soluble FasL could inhibit FasL-mediated cytotoxicity [33]. Third, as *L. pifanoi* and *L. amazonensis* block macrophage apoptosis through activation of PI3K/Akt pathway [34], *L. major* could induce anti-apoptotic signaling more efficiently in B6 macrophages. Our results suggested that FasL does not have a role in the initial stages of *Leishmania* infection in resistant mice.

Infection with *L. major* increased the secretion of TNF-α, IL-6, TIMP-1, IL-1RA, G-CSF and TREM, but not IL-1α or IL-1β. In addition, infection increased secretion of chemokines KC, MIP-1α, MIP-1β, MCP-1 and MIP-2. Our results agreed with previous reports of increased expression of TNF-α, MIP-1α, MIP-1β, MIP-2, MCP-1 and KC [6], [7]. One study also identified increased gene expression for IL-1RA and unchanged gene expression for IL-1β [7]. Our studies were done at the protein level, and found that infection did not induce secretion of IL-1β by resident macrophages. However, it is likely that IL-1 can be produced by other phagocytes [35]. The role of IL-1RA is unclear, since IL-1 is dispensable for protection of B6 mice [35]. Furthermore, our results showed that infection increased TIMP-1 expression by resident macrophages, and we also found increased metalloproteinase expression following infection (data not shown). These results are relevant for disease, since expression of TIMP-1 (a tissue inhibitor of metalloproteinases) could be involved in the regulation of metalloproteinase activity, tissue damage and spread of infection in cutaneous and visceral leishmaniasis [36], [37]. Our results also indicated a sentinel role for resident macrophages, through release of several chemokines upon infection with *L. major*.

Infection activated the JNK pathway and induced secretion of the chemokine KC (CXCL1). Secretion of KC was completely blocked by the JNK inhibitor SP600125, and partially blocked by the ERK inhibitor SB203580, but not by p38 inhibitor PD98059. These results suggested that JNK pathway, and ERK pathway to a lesser extent, are involved in chemokine secretion induced by infection. JNK inhibitor SP600125 also inhibited parasite replication in macrophages, suggesting a role for JNK in intracellular parasite survival and growth. It is interesting that JNK both induces inflammatory signals like KC and promotes parasite growth. However, *Leishmania* parasites express homologues of mammalian MAPK [38]. Therefore, we cannot discard that the JNK inhibitor could affect the parasite directly. Further studies are necessary to identify the mechanisms by which JNK inhibitor blocks the intramacrophage replication of the parasite. Oxidative stress activates the JNK pathway [21]–[23]. Our results demonstrated that, besides blocking ROS generation, antioxidants DFO and NAC partially decreased JNK activation and reduced KC secretion induced by infection. Taken together, these results suggested that infection triggers an intracellular pathway that sequentially recruits ROS, JNK and KC. In agreement with the anti-parasite effects of the JNK inhibitor, DFO and NAC potently inhibited intracellular parasite replication in macrophages. Our data agree with the recently identified role of ROS in intracellular survival/growth of *Leishmania* and *Trypanosoma cruzi* parasites [4], [5], [39]. However, it should be noted that ROS inhibitors induced a more potent blockade in parasite replication than in JNK activation. ROS could have direct effects on parasite replication and additional indirect effects besides activation of the JNK pathway. For example, ROS are critically involved in M2-type macrophage differentiation [40]. In agreement with this possibility, neutrophil elastase, a potent ROS inducer, promotes M2-type differentiation, which favors replication of *L. major* in macrophages [41].

In conclusion, infection with *L. major* induces a cellular stress response in tissue resident macrophages. The stress response includes ROS generation and activation of the JNK/c-Jun/FasL cascade, leading to chemokine secretion and increased parasite survival. How this stress response is generated remains to be investigated. Sustained movement of *L. donovani* parasites inside macrophages leads to plasma membrane wounding and repair through lysosomal exocytosis [42]. Membrane wounding could be the stimulus for triggering a stress response. Interestingly, infection of macrophages with *L. donovani* generates ceramide [43], which is known to activate the SAPK/JNK pathway [12], and is required for parasite survival [43]. Together, these and our results suggest new targets for therapeutic intervention in leishmanial infection.

## Materials and Methods

### Ethics Statement

This study was carried out in strict accordance with the recommendations in the Guide for the Care and Use of Laboratory Animals of the National Institutes of Health (USA). The protocol was approved by the Committee on the Ethics of Animal Experiments of the Health Science Center of the Federal University of Rio de Janeiro (CEUA-CCS, Permit Number: IBCCF 178) and all efforts were made to minimize suffering.

### Antibodies and Chemicals

Dulbecco’s Modified Eagle’s Medium (DMEM), RPMI medium, fetal calf serum (FCS), glutamine, gentamicin, sodium pyruvate, MEM nonessential amino acids, and HEPES buffer were obtained from GIBCO/Invitrogen. The following reagents were used: Nutridoma SP (Roche); propidium iodide, DMSO, PE-conjugated Annexin V, hamster IgG1/κ, PE- and FITC-conjugated hamster anti-mouse Fas and FasL monoclonal antibodies (mAbs), APC/Cy7-conjugated anti-Ly6G, purified hamster anti-FasL MFL3 mAb, hamster IgG1 isotype control, anti-CD16/CD32 mAb (FcBlock), all from BD Biosciences; PerCP/Cy5.5-conjugated anti-Ly6C mAb HK1.4 (eBioscience); rabbit SAPK/JNK mAb 56G8; phospho-SAPK/JNK (Thr183/Tyr185) mAb 81E11; anti-c-Jun mAb 60A8, phospho-c-Jun-Ser63 mAb 54B3, Goat anti-rabbit IgG, HRP-conjugated (Cell Signaling); JNK inhibitor SP600125 (Enzo Life Sciences), ERK (MEK) inhibitor PD98059, p38 inhibitor SB203580 (EMD Millipore).

### Mice and Parasites

C57BL/6 (B6) and BALB/c mice were from Oswaldo Cruz Institute, Rio de Janeiro, Brazil. For flow cytometry experiments, wild-type B6 and FasL-deficient mutant *gld* B6 mice were obtained from The Jackson Laboratory. All animal work was approved and conducted according institutional guidelines. *Leishmania major* (*L. major*) strain LV39 (MRHO/Sv/59/P) was isolated from BALB/c mice, and maintained *in vitro* for up to 4 wk [44]. Parasites were maintained in Schneider medium (Life Technologies) supplemented with 10% FCS, 1% glutamine and 2% human urine [8], [45].

### Macrophages and Infection

Resident macrophages were obtained by washing the peritoneal cavity of B6 mice and discarding nonadherent cells after 20 h culture. Inflammatory macrophages were obtained 4 d after i.p. injection of 1 ml of 3% thioglycollate broth (Sigma-Aldrich). Nonadherent cells were discarded after 4–20 h. Resident (2×10^5^) and inflammatory cells (1.5×10^5^) were cultured on 48-well plates (Nunc, Denmark), yelding approximately 1×10^5^ adherent macrophages. Cultures were done in 0.5 ml supplemented DMEM medium containing 10% FCS or 1% Nutridoma. DMEM and RPMI media were supplemented with glutamine, 2-ME, gentamicin, sodium pyruvate, MEM nonessential amino acids, HEPES buffer, and 10% FCS or 1% Nutridoma. Adherent macrophages were infected with *L. major* promastigotes at 10∶1 parasite/macrophage ratio for 4–20 h at 37°C and 7% CO_2_.

### Assessment of Parasite Load

For assessment of parasite internalization, resident and inflammatory macrophage monolayers (10^5^ adherent cells) were established in glass coverslips and infected in replicates with *L. major* promastigotes at a 10∶1 parasite/cell ratio. After 4 h at 37°C, extracellular parasites were washed. Coverslips were stained by Romanowsky stain, and both percentage of infected macrophages and parasite number per 100 macrophages were determined. For assessment of parasite load after 3 d of infection, resident macrophages (1×10^5^) were treated with either solvent or inhibitors 3 h prior to infection, and infected with 1×10^6^ *L. major* promastigotes in DMEM supplemented with 10% FCS or 1% Nutridoma in 48-well vessels. After 20 h, extracellular parasites were removed, and macrophages were cultured with medium containing 10% FCS or 1% Nutridoma in the continued presence of the inhibitors for additional 3 d. All cultures were washed and transferred to Schneider medium (GIBCO-Invitrogen) supplemented with 20% FCS, 1% glutamine, 2% human urine, as described [8], [43], and maintained at 26°C for additional 3 d. The relative intracellular load of *L. major* was assessed by counting the number of motile extracellular promastigotes released in each well [8], [45].

### Detection of ROS

Intracellular levels of ROS were measured by oxidation of nonfluorescent 2′, 7′ dichlorofluorescin probe, delivered as diacetate form (DCFH-DA), to the fluorescent product 2′,7′ dichlorofluorescein [46]. Resident and inflammatory macrophages (10^5^ cells) were prepared in 96-well plates, and loaded for 10 min at 37°C with 10 µM DCFH-DA (Sigma-Aldrich). Macrophages were washed and infected with *L. major* for 4 h. In preliminary kinetic experiments, this time of infection gave the strongest signal for this parasite isolate. Macrophages were washed again and fluorescence was measured (485 nm excitation; 535 nm emission) after 60 min in an FLx800 Fluorescence Microplate Reader (BioTek).

### Western Blot Analysis

Macrophages were infected or not with *L. major*, washed and incubated for 4 h. For detection of metalloproteinases, macrophages were incubated for 48–72 h. After culture, cells were washed in PBS and treated with ice-cold RIPA lysis buffer containing protease inhibitor cocktail and sodium orthovanadate 2 mM for 20 min. The suspensions were collected, homogenized and centrifuged in 10,000 g for 20 min at 4°C. The supernatants were obtained and the protein content evaluated by the Bradford method. Proteins were precipitated with 90% ice-cold ethanol in order to obtain equal amounts of protein samples. Protein samples (80 µg/lane) were suspended in 2X Laemmli sample buffer and 5% β-mercaptoethanol. After boiling for 5 min, samples were separated in 10% acrylamide SDS-PAGE, and electrotransferred to nitrocellulose membranes (Hybond-C, Amersham Biosciences) using the Bio-Rad mini vertical Trans-Blot Cell system. Blots were blocked with TBS-3% BSA for 1 h at room temperature, and incubated with the primary antibodies (1∶1,000) in TBS-3% BSA-0.05% sodium azide for 18 h at 4°C. Blots were then washed 3 times with TBS-0.05% Tween-20 for 10 min, once with TBS for 5 min at room temperature, and then incubated with HRP-conjugated (1∶2,000), IRDye 680LT or IRDye 800CW-conjugated (1∶10,000) secondary antibodies in TBS-3% BSA for 1 h at room temperature. After one more wash cycle, the antibodies were detected with ECL Plus Western Blotting detection system, GE Healthcare Life Sciences (Pittsburgh, PA) or by fluorescence imaging using a Li-Cor Odyssey infrared scanner. The images were processed using Adobe Photoshop CS and ImageJ softwares. Protein loading was verified by either red Ponceau staining or immunoblotting. For densitometric analysis, blots were scanned, and intensities of bands were quantitated with ImageJ software. The area of both p54 and p46 bands of p-JNK were measured. Intensities were normalized as percentages of the maximum values (total JNK staining).

### Flow Cytometry

Adherent macrophages were detached by treatment with PBS-5 mM EDTA-1% FCS, washed in staining buffer containing 2% FCS, and incubated with anti-CD16/CD32 (Fc block). Cells were stained with antibodies for 30 min at 4°C. Cells were gated for F4/80^+^ CD11b^+^7AAD^−^ viable macrophages, and analysed for staining with PE-Annexin V and FITC-anti-FasL. Cells were acquired on a FACSCalibur^™^ flow cytometer, by using Cell quest software (BD Biosciences). For analysis, FlowJo^™^ software was used (TreeStar).

### Cell Viability Assay

Macrophage viability was determined by lysozyme activity in culture supernatants. Lysozyme is a marker of macrophage viability [47]. Lysozyme activity was measured by fluorescence quenching of fluorescently labeled *Micrococcus lysodeikticus* cell walls (EnzChek^™^ Lysozyme Assay Kit; Life Technologies). As a negative control, macrophages were fixed with 4% paraformaldehyde (PFA) to measure residual lysozyme activity.

### Production of Cytokines and Chemokines

Supernatants from either uninfected or infected macrophages were simultaneously assayed for 40 different cytokines and chemokines using the Proteome Profiler^™^ Mouse Cytokine Antibody Array Kit, according to the manufacturer instructions (R&D Systems). The densities of individual stainings were compared with image processing software ImageJ 1.46 r. Data are mean and SE of duplicates. In addition, supernatants were assayed for soluble FasL, IL-1β, IL-6, KC, and TNF-α by sandwich ELISA (R&D Systems). Results are mean and SE of triplicate cultures.

### Statistical Analysis

Quantitative data shown are representative experiments involving pools at 2–3 animals, and were repeated at least three times, with similar results. Data represent mean and SE of triplicates, at least. Data were analysed by Student’s *t* test for independent samples, using SigmaPlot™ for Windows. Differences with a *P* value < 0.05 or lower were considered significant. Proteomic profiling was repeated twice with similar results. Densitometric data of readings were normalized as percentage of a positive control provided by the kit. Data were compared in quadruplicates using non-parametric Mann-Whitney U test. Cytokines which gave a significant increase (*P*<0.05 or lower) following infection are marked by an asterisk.
